# Special Issue: Novel Approaches for the Analytical Evaluation of Food Quality and Authenticity

**DOI:** 10.3390/foods12193651

**Published:** 2023-10-03

**Authors:** Vito Gallo, Biagia Musio

**Affiliations:** Department of Civil, Environmental, Land, Building Engineering and Chemistry (DICATECh), Polytechnic University of Bari, I-70125 Bari, Italy; vito.gallo@poliba.it

Verifying the quality and authenticity of agri-food products is essential to guaranteeing adequate food safety for consumers. Ideally, the traceability of food products should be ensured throughout the entire supply chain, i.e., from raw materials to the final product, and vice versa. To achieve this objective, it is crucial to detect and quantify specific analytes which, if contained in food products, can act as indicators of authenticity. Nowadays, there is increasing interest in the implementation of new analytical methods characterized by the properties of high sensitivity, high throughput, and easy validation. Analytical results should be directly interpreted through mathematical models that are capable of providing rapid and reliable information regarding the quality and authenticity of the food product under study [1,2].

This Special Issue will focus on the most recent advances in this field, exploring the application of modern analytical techniques to assess the quality of food products. For this purpose, different analytical techniques, used individually or in combination, will be taken into consideration [3]. Furthermore, scientific works on artificial intelligence-oriented methods applied to food control will be welcome. These should move from chemometrics to machine learning, and deep learning [4].

Moreover, this Special Issue will compile scientific works on the applications of targeted and non-targeted metabolomics for the quality control of food products and their traceability through the supply chain [5,6]. The targeted analyses should aim at identifying specific metabolites the presence of which is indicative of a peculiar property of the sample. The quantification of such substances and the comparison of their amounts with established reference values should be in accordance with validated and standardized procedures. Nevertheless, while the targeted approach is excellently employed for the detection and quantification of known compounds in a complex matrix, it is often lacking in applicability for identifying unknown additives or contaminants that may hamper the authenticity and quality of a product. In this context, studies on non-targeted approaches are welcome. These should provide a comprehensive description of the complex matrix under investigation, enabling the detection of both known and unknown compounds contained in the mixture. The resulting indications of samples should be primarily intended not only for the identification of analytes but also for the assessment of sample authenticity via the use of suitable classification tools [7].

As an element of this Special Issue, our goal is to promote research and development in the adoption of a new scientific mindset that exploits innovative and smart technologies for food analysis. We welcome both original research and high-quality review articles in this interdisciplinary field.

Potential topics may include but are not limited to the following:

- Smart analytical technologies to improve the traceability of the supply chain, and also for single steps from production to the final consumer;
- The development and validation of real-time analytical methods to test the authenticity and quality of food products;
- Tests on raw materials to guarantee the safety and quality of food products;
- Tools to identify and quantify nutraceuticals within food extracts;
- Statistical tools and approaches to validate targeted and non-targeted analytical procedures;
- Tracking methods adopting emerging technologies, such as blockchain and AI-oriented tools.
